# Uncovering Urinary Neonicotinoid Exposure Signatures Among Older Adults in South China: A Multicenter Biomonitoring Study

**DOI:** 10.3390/toxics14070641

**Published:** 2026-07-22

**Authors:** Xiaoxiao Chen, Chiqun Shan, Yiming Ge, Yuli Lin, Bo Fu, Canrong Zheng, Yuhua Huang, Shaoyou Lu

**Affiliations:** 1 Chaozhou People’s Hospital, Chaozhou Hospital Affiliated to Shantou University Medical College, Chaozhou 521011, China; 15992360101@163.com (X.C.); 13715821998@163.com (C.Z.); 2 Research Center for Environment and Health, School of Public Health (Shenzhen), Sun Yat-sen University, Shenzhen 518107, China; shanchq3@mail2.sysu.edu.cn (C.S.); geym5@mail2.sysu.edu.cn (Y.G.); linyli6@mail2.sysu.edu.cn (Y.L.); fubo7@mail2.sysu.edu.cn (B.F.)

**Keywords:** neonicotinoids, urinary biomarkers, older adults, biomonitoring, South China

## Abstract

Neonicotinoid insecticides (NEOs) are widely used neuroactive pesticides, but biomonitoring evidence among older adults remains limited. This multicenter cross-sectional study characterized urinary exposure profiles of NEOs and their metabolites among 419 older adults from South China. Ten urinary NEO biomarkers, including six parent compounds and four metabolites, were measured using high-performance liquid chromatography coupled with tandem mass spectrometry. NEOs were frequently detected in urine samples; except for thiacloprid and acetamiprid, with detection frequencies of 81.67% and 65.48%, respectively, all other compounds were detected in more than 90% of participants. Among the parent NEOs, clothianidin had the highest median concentration (1.402 μg/L), followed by dinotefuran (1.240 μg/L) and thiamethoxam (0.888 μg/L). The main metabolite was *N*-desmethyl-acetamiprid (1.643 μg/L). Looking at the overall composition, dinotefuran, clothianidin, thiamethoxam, and *N*-desmethyl-acetamiprid were the biggest contributors to the total urinary NEO burden, though their shares varied by region and recruitment center. Correlation analysis pointed to common co-exposure patterns, especially between thiamethoxam and clothianidin, and between imidacloprid and its metabolites. In exploratory analyses, *N*-desmethyl-thiamethoxam showed a positive correlation pattern, with significant positive correlations with glucose, glycated hemoglobin, triglycerides, and alanine aminotransferase (all *q* < 0.01). CLO was positively correlated with high-density lipoprotein cholesterol (*q* < 0.05). These findings indicate widespread co-exposure to multiple NEOs among older adults in South China and highlight the need for longitudinal studies to clarify exposure sources, temporal variability, and potential health implications.

## 1. Introduction

Neonicotinoid insecticides (NEOs) are nicotine-related neuroactive pesticides that act mainly on insect nicotinic acetylcholine receptors [1,2]. Owing to their high efficacy and broad applicability, NEOs have become one of the major classes of insecticides used worldwide [3]. In China, their use is also substantial. For example, an estimated 1361 tons of NEOs were applied in the Pearl River Basin in 2019 [4]. Moreover, because of their water solubility and environmental persistence, NEOs are widely present in soil, water, and other environmental media [5,6,7]. Humans can be exposed to NEOs through multiple pathways, including dietary intake, inhalation, and dermal contact [2]. NEOs and their metabolites have been widely detected in various human biological matrices, including urine, serum, hair, and breast milk [8,9,10,11]. Owing to the relatively short biological half-lives of NEOs and the non-invasive nature of urine collection, urine is the most commonly used matrix for assessing human exposure to NEOs [2,12].

Although NEOs were initially considered to have relatively low toxicity to mammals, growing evidence suggests that they may be associated with potential health risks [1,13]. Behavioral alterations induced by NEO exposure have been observed in various non-target organisms, including honey bees [14] and hummingbirds [15]. Experimental studies conducted by Hellfeld [16] and Hawkey [17] using zebrafish models, as well as by Koike [18] in mice, consistently demonstrated the neurotoxic potential of NEOs. In addition to neurotoxicity, reproductive and developmental toxicity [19,20], immunotoxicity [21,22], and genotoxicity [23] have also been reported in animal studies. Similar findings have increasingly emerged from epidemiological studies in human populations. Borkar et al. systematically reviewed the findings from 35 epidemiological studies and concluded that NEO exposure was associated with neurotoxicity [24]. In addition, Deng et al. measured urinary NEOs and metabolites in 225 healthy adults and found significant positive associations between NEO exposure and oxidative stress biomarkers [25]. Emerging evidence also suggests the association between NEO exposure and increased risk of obesity [26,27]. Moreover, Guo et al. reported that NEOs, together with metal co-exposure, were associated with thyroid hormone disruption in children from a rural area in South China [28]. In adults, analyses based on the National Health and Nutrition Examination Survey (NHANES) 2015–2016 indicated that detectable urinary NEO biomarkers were associated with liver function-related indicators, although the direction and strength of associations varied by biomarker and sex [29].

However, existing epidemiological studies have largely focused on specific vulnerable populations, such as children and pregnant women, or on general adult populations, whereas data on older adults remain limited. Older adults may be especially vulnerable to environmental chemicals because aging is associated with altered metabolism and excretion, reduced renal and hepatic clearance, chronic disease burden, and longer cumulative environmental exposure histories [30,31,32]. Although recent Chinese studies have associated urinary NEO exposure with dyslipidemia and hypertension among elderly populations, systematic evidence on exposure profiles in older adults remains scarce [3,33]. Considering the intensive agricultural activities, high population density, and diverse dietary habits in South China, further investigation is warranted to characterize NEO exposure and its variation across demographic, lifestyle, and clinical characteristics in older adults [34,35].

Therefore, the present multicenter study aimed to characterize urinary NEOs among older adults from Guangzhou and Shenzhen, Guangdong Province, South China. Specifically, this study sought to: (1) determine the detection frequencies and concentrations of urinary NEOs and their metabolites; (2) describe the composition profiles and distribution patterns of urinary NEOs overall and across different study regions; and (3) explore differences in NEO exposure according to demographic, lifestyle, and clinical characteristics, as well as potential factors associated with urinary NEO concentrations.

## 2. Materials and Methods

### 2.1. Chemicals and Reagents

Thiacloprid (THD), thiamethoxam (THM), clothianidin (CLO), imidacloprid (IMI), acetamiprid (ACE), and dinotefuran (DIN) were purchased from Anpel Laboratory Technologies Inc. (Shanghai, China). 5-Hydroxy-imidacloprid (5-OH-IMI) was obtained from Witega (Berlin, Germany). Imidacloprid-olefin (IMI-OF), *N*-desmethyl-acetamiprid (DM-ACE), and *N*-desmethyl-thiamethoxam (DM-THM) were purchased from Dr. Ehrenstorfer GmbH (Augsburg, Germany). The isotope-labeled internal standards, including thiacloprid-D4 (THD-D4), thiamethoxam-D3 (THM-D3), clothianidin-D3 (CLO-D3), imidacloprid-D4 (IMI-D4), acetamiprid-D3 (ACE-D3), and dinotefuran-D3 (DIN-D3), were purchased from ALTA Scientific Co., Ltd. (Tianjin, China). β-Glucuronidase, methanol (≥99.9%), and formic acid (≥98.0%) were also obtained from Anpel Laboratory Technologies Inc. (Shanghai, China). Acetonitrile (≥99.9%), ethyl acetate (≥99.8%), ammonium acetate, and acetic acid (≥80.0%) were obtained from Macklin Biochemical Co., Ltd. (Shanghai, China).

### 2.2. Sample Collection

This multicenter cross-sectional study included older adults recruited from five hospitals and community health service centers in Guangzhou and Shenzhen, Guangdong Province, South China, between January and August 2023 (Table S1). Participants were enrolled during routine health examinations or community-based health management programs. Individuals aged 60 years or older who provided sufficient urine samples and complete demographic, lifestyle, and basic clinical information were eligible for inclusion. Participants with insufficient urine volume, incomplete key information, severe renal dysfunction, or malignant diseases were excluded from the final analysis. A total of 419 older adults were included in the final analysis.

First-morning spot urine samples were collected by trained staff in polyethylene centrifuge tubes, sealed, and stored at −20 °C within 2 h of collection until laboratory analysis. Face-to-face questionnaires and clinical examinations were conducted concurrently. Demographic characteristics, lifestyle factors, and clinical information, including age, sex, smoking status, alcohol consumption, hypertension history, fasting blood glucose (GLU), glycated hemoglobin (HbA1c), serum uric acid (SUA), serum creatinine (SCr), total cholesterol (TC), triglycerides (TG), low-density lipoprotein cholesterol (LDL), high-density lipoprotein cholesterol (HDL), alanine aminotransferase (ALT), and aspartate aminotransferase (AST), were obtained from standardized questionnaires, medical records, or clinical examinations.

All participants were informed of the study objectives and procedures and provided written informed consent prior to participation. The study protocol was approved by the Ethics Committee of the School of Public Health (Shenzhen), Sun Yat-sen University (approval number: 2022015).

### 2.3. Determination of Urinary NEOs and Metabolites

The sample pretreatment and instrumental analysis procedures used in this study were based on a previously reported method [36]. The detailed protocols are provided in Text S1 and Table S2. Briefly, urine samples were enzymatically hydrolyzed and incubated for 12 h to release conjugated metabolites. After hydrolysis, target analytes were extracted using liquid–liquid extraction with ethyl acetate, followed by instrumental analysis. All analytes were quantified by high-performance liquid chromatography (HPLC, Shimadzu 20A, Kyoto, Japan) coupled with a triple quadrupole mass spectrometer (QTRAP 6500, Applied Biosystems, Waltham, MA, USA). Information on quality assurance and quality control is available in Text S2 and Table S3.

### 2.4. Statistical Analysis

Concentrations of NEOs and their metabolites below the limits of detection (LODs) were replaced with LOD/√2. Detection frequency (DF) was defined as the proportion of samples with concentrations above the LOQ. ΣNEOs was calculated as the sum of the measured parent NEOs and metabolites after substitution of values below the LOD.

Participant characteristics were summarized for the total population and separately for Guangzhou and Shenzhen. Continuous variables were presented as medians and interquartile ranges (IQRs), and categorical variables were expressed as numbers and percentages. Differences between Guangzhou and Shenzhen were evaluated using the Mann–Whitney U test for continuous variables and the chi-square test or Fisher’s exact test for categorical variables, as appropriate. Given the right-skewed distributions of urinary NEO concentrations, exposure levels were summarized using arithmetic means with standard deviations (SDs), geometric means with geometric standard deviations (GSDs), medians with IQRs, and the 95th percentile (P95). Urinary NEO concentrations were log-transformed before correlation and regression analyses.

Spearman correlation analysis was used to assess correlations among urinary NEO biomarkers and between urinary NEO biomarkers and continuous participant characteristics or clinical indicators, including age, GLU, HbA1c, SUA, SCr, TC, TG, LDL, HDL, ALT, and AST. To evaluate associations between categorical participant characteristics and urinary NEO biomarkers, multivariable linear regression models were fitted with log-transformed urinary NEO concentrations as dependent variables. Each model included one categorical characteristic of interest (sex, smoking status, alcohol consumption, or hypertension status) as the independent variable, and was adjusted for age, study center, SCr, and the remaining three categorical characteristics. For the analyses involving exposure–outcome associations in both correlation and regression analyses, False Discovery Rate (FDR) correction using the Benjamini–Hochberg method was applied to account for multiple comparisons. Heatmaps were used to visualize Spearman correlation coefficients for continuous variables and multivariable-adjusted regression coefficients (β) for categorical variables. All analyses were performed using R4.4.3. A two-sided *p* < 0.05 was considered statistically significant, and associations with an FDR-adjusted *q* < 0.05 were regarded as robust.

## 3. Results

### 3.1. Participant Characteristics

A total of 419 older adults were included in this study, comprising 140 participants from Guangzhou and 279 participants from Shenzhen (Table 1). Overall, 53.2% of participants were male and 46.8% were female. The median age of the study population was 66 years (IQR: 63–72 years).

No significant difference in sex distribution was observed between Guangzhou and Shenzhen. However, participants from Shenzhen were significantly older than those from Guangzhou (median: 68 vs. 65 years, *p* < 0.001). The proportions of smokers and alcohol drinkers were also significantly higher in Shenzhen than in Guangzhou, with smoking rates of 22.6% versus 11.4% (*p* < 0.001) and alcohol consumption rates of 13.6% versus 2.9% (*p* < 0.001), respectively.

The prevalence of hypertension was significantly higher in Shenzhen than in Guangzhou (37.3% vs. 22.9%, *p* = 0.004). Regarding metabolic indicators, participants from Shenzhen had significantly higher levels of GLU, HbA1c, SUA, TG, and ALT than those from Guangzhou (all *p* < 0.05). In contrast, SCr levels were slightly higher in Guangzhou than in Shenzhen (*p* = 0.011). No statistically significant regional differences were observed for TC, LDL, HDL, or AST levels.

### 3.2. Detection Frequencies and Concentrations of Urinary NEOs and Metabolites

The detection frequencies and urinary concentrations of NEOs and their metabolites were shown in Table 2. Overall, NEOs were frequently detected in urine samples from older adults. Except for THD and ACE, which showed relatively lower detection frequencies of 81.67% and 65.48%, respectively, all other compounds were detected in more than 90% of participants.

Regarding exposure levels, the median concentration of ΣNEOs was 10.928 μg/L (IQR: 6.170–19.620), with a P95 concentration of 41.624 μg/L. Among the parent NEOs, CLO showed the highest median concentration (1.402 μg/L), followed by DIN (1.240 μg/L) and THM (0.888 μg/L). THD and ACE showed the lowest median concentrations, at 0.018 μg/L and 0.025 μg/L, respectively. Among the metabolites, DM-ACE was the predominant compound, with a median concentration of 1.643 μg/L, whereas DM-THM showed the lowest median concentration (0.184 μg/L). The P95 concentrations were much higher than the median values for several compounds, particularly DIN (12.149 μg/L), DM-ACE (9.936 μg/L), CLO (9.318 μg/L), THM (6.506 μg/L), and IMI-OF (6.300 μg/L), indicating relatively high exposure levels in a subset of participants.

To further contextualize our findings, urinary NEO concentrations were compared with recent biomonitoring studies from different countries and populations (Table 3). The exposure profile in the present study was broadly comparable to recent studies from South China, particularly those of elderly adults and general adults, in which DIN, CLO, IMI-related metabolites, and DM-ACE were also major urinary biomarkers [3,25]. In contrast, studies among pregnant women from East China and Tibet generally reported lower median concentrations, with many parent compounds below the LOD, although DM-ACE and CLO were still detected in some participants [37,38]. Compared with studies from Ireland and the Philippines, the present study showed higher median concentrations and broader detection of multiple parent NEOs and metabolites [39,40]. These differences may reflect variations in age structure, dietary habits, residential environment, pesticide use patterns, regional contamination, analytical methods, and urine dilution correction.

### 3.3. Composition Profiles of Urinary NEOs and Metabolites

**Figure 1:**
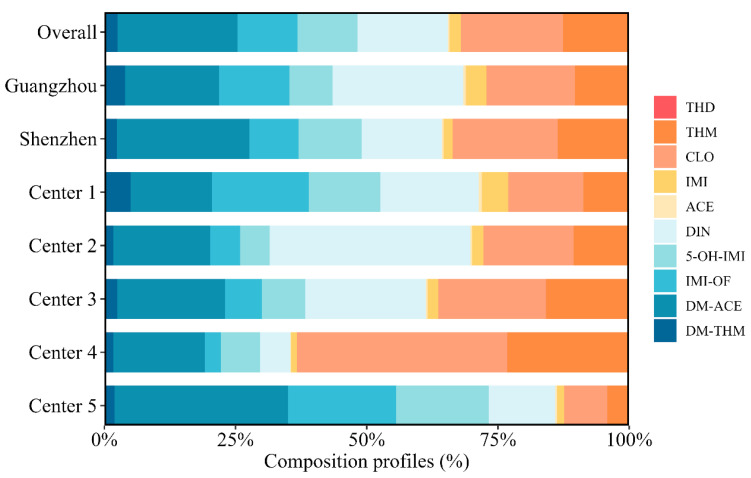
Composition profiles of urinary NEOs and metabolites overall and stratified by region and study center. The stacked bars show the relative contribution of each NEO biomarker to the total measured urinary NEO burden. The overall profile is presented for all participants, followed by profiles stratified by region and study center.

Figure 1 presents the composition profiles of urinary NEOs in the overall population and across regional and center-specific groups. Overall, the urinary NEO profile was dominated by several compounds, with CLO, DIN, THM, and DM-ACE contributing relatively large proportions to the total NEO burden. In contrast, some parent compounds or metabolites, such as THD and 5-OH-IMI, accounted for relatively small proportions in most groups.

At the regional level, Guangzhou and Shenzhen showed broadly similar overall composition patterns, although differences in the relative contributions of individual compounds were observed. Compared with Guangzhou, Shenzhen had a higher relative contribution of DM-ACE but a lower contribution of DIN. Center-specific profiles further showed some heterogeneity across the five study centers. Center 2 was dominated by DIN, whereas Center 4 showed much higher contributions of CLO and THM. In contrast, Center 5 had the highest relative contributions of DM-ACE and IMI-OF. Centers 1 and 3 showed relatively balanced profiles, with DIN, DM-ACE, CLO, and THM all contributing appreciably. These differences indicate that urinary NEO compositions varied by recruitment site, possibly reflecting differences in dietary habits, environmental exposure, pesticide use patterns, or participant characteristics.

### 3.4. Correlation Analysis Among Urinary NEOs and Metabolites

**Figure 2:**
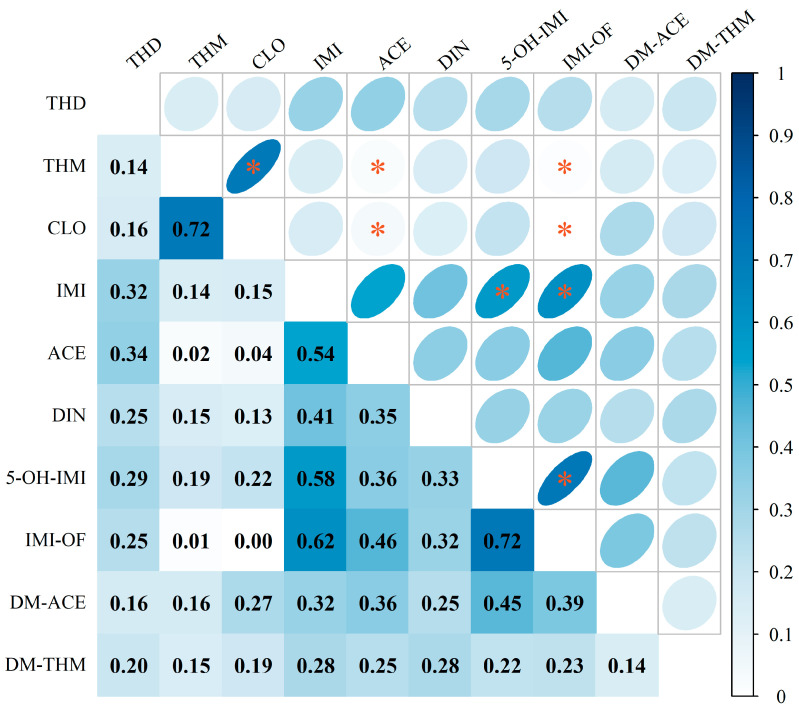
Spearman correlation heatmap among urinary NEOs and metabolites. Spearman correlation coefficients were calculated using log-transformed urinary concentrations of NEOs and metabolites. The color scale represents the magnitude and direction of the correlation coefficients. Asterisks indicate statistical significance: * *p* < 0.05, ** *p* < 0.01, *** *p* < 0.001.

The relationships among urinary concentrations of individual NEOs and their metabolites are presented in Figure 2. Overall, most compounds showed positive correlations with each other, indicating that older adults were commonly exposed to multiple NEOs simultaneously.

Relatively strong correlations were observed between THM and CLO (r = 0.72), suggesting that these two compounds may have shared exposure sources or similar environmental pathways. IMI was also positively correlated with its metabolites, including 5-OH-IMI (r = 0.58) and IMI-OF (r = 0.62), while 5-OH-IMI and IMI-OF were strongly correlated with each other (r = 0.72). These findings support the consistency of IMI-related metabolic patterns in urine.

Several moderate correlations were also identified, including IMI with ACE (r = 0.54), ACE with IMI-OF (r = 0.46), and 5-OH-IMI with DM-ACE (r = 0.45). However, some compound pairs showed weak or negligible correlations, such as THM with ACE (r = 0.02) and CLO with IMI-OF (r < 0.01).

### 3.5. Associations Between Urinary NEOs and Metabolites with Potential Influencing Factors

**Figure 3:**
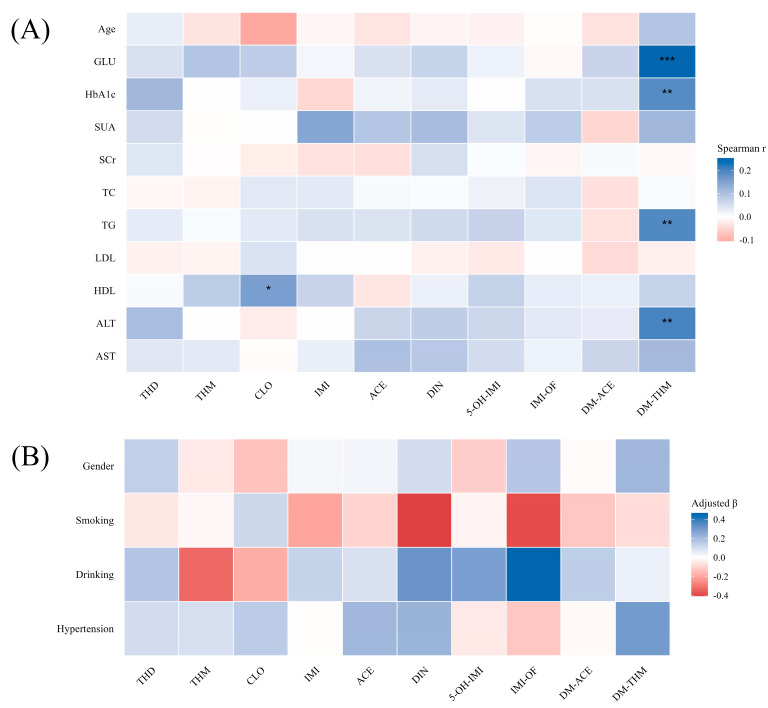
Associations of urinary NEO exposure with clinical indicators and participant characteristics. (**A**) Spearman correlations between log-transformed urinary NEO concentrations and continuous variables, including age and clinical indicators. (**B**) Regression coefficients from multivariable linear models assessing associations between categorical participant characteristics and log-transformed urinary NEO concentrations, adjusted for age, sex, smoking status, alcohol consumption, hypertension status, study center and SCr. Color intensity represents Spearman correlation coefficients (r) in panel (**A**) and regression coefficients (β) in panel (**B**). Blue indicates positive associations and red indicates negative associations. Statistical significance is based on false discovery rate (FDR)-adjusted *q* values: * *q* < 0.05, ** *q* < 0.01, *** *q* < 0.001.

Exploratory associations between urinary NEO biomarkers and participant characteristics or clinical indicators were evaluated using Spearman correlation analysis and multivariable linear regression. As shown in Figure 3A, several urinary NEO biomarkers were significantly associated with continuous variables. DM-THM showed the most consistent and robust correlation pattern, with significant positive correlations with GLU, HbA1c, TG, and ALT (all *q* < 0.01). CLO was positively correlated with HDL (*q* < 0.05). Several additional correlations were nominally significant before correction, including positive correlations of THD with HbA1c and ALT, a negative correlation of CLO with age, positive correlations of IMI and DIN with SUA, and a positive correlation of ACE with AST (all *p* < 0.05) (Table S4). However, these associations did not remain significant after FDR correction and should therefore be regarded as exploratory.

Figure 3B further shows the associations of urinary NEOs with demographic characteristics, lifestyle factors, and hypertension status. In these adjusted models, the directions of several associations were consistent with those observed in unadjusted analyses (Table S5). For example, alcohol consumption tended to be inversely associated with THM, and hypertension tended to be positively associated with ACE and DM-THM. However, after FDR correction none of the associations between categorical characteristics and urinary NEO biomarkers remained statistically significant (all *q* > 0.05).

## 4. Discussion

In this multicenter biomonitoring study, urinary NEOs and their metabolites were widely detected among older adults from Guangzhou and Shenzhen. Except for THD and ACE, with detection frequencies of 81.67% and 65.48%, respectively, all other compounds were detected in more than 90% of participants, indicating pervasive exposure to multiple NEO compounds in South China. Compared with biomonitoring studies from other countries and regions, our participants showed a broader and more frequent detection profile. In NHANES 2015–2016, 49.1% of participants had at least one detectable urinary NEO biomarker, mainly DM-ACE and 5-OH-IMI [42]. In a Japanese study, DM-ACE, CLO, DIN, and THM displayed high detection frequencies (all > 90%), with DM-ACE detected in 100% of samples. However, ACE, THD, and DM-THM showed much lower detection rates, all falling below 25% [43]. In an Irish family-based study, although at least one NEO biomarker was quantified in 75% of urine samples, most individual parent compounds were infrequently detected or below quantification limits [40].

Several factors may contribute to these elevated exposure levels. First, South China, particularly the Pearl River Basin and Pearl River Delta, is characterized by intensive agricultural activities, rapid urban–rural development, and favorable climatic conditions for year-round crop production, which together may increase pesticide demand and facilitate environmental dissemination of NEOs [4]. Previous environmental monitoring studies have detected widespread NEO contamination in surface water, sediments, soils, and agricultural products within the Pearl River Basin [44,45]. Environmental NEOs may enter the human body through multiple pathways, with dietary intake considered one of the major exposure routes for the general population, particularly through the consumption of vegetables, fruits, and tea products containing pesticide residues [46,47].

Second, the high detection frequencies observed in our study may also be related to the characteristics of older adults. Consistent with our findings, previous studies in elderly Chinese populations have also reported widespread urinary NEO exposure. Tang et al. found that, except for IMI, all measured NEOs and metabolites were detected in more than 80% of 1147 older adults in Shenzhen, while Sun et al. reported detection frequencies of 87.2–99.6% among 500 community-dwelling elderly adults in China [3,33]. From a toxicokinetic perspective, aging-related changes in renal clearance, hepatic metabolism, body composition, and chronic disease burden may influence the absorption, metabolism, and urinary excretion of xenobiotics, thereby partly affecting urinary NEO biomarker profiles [31,32]. However, this interpretation should be confirmed in longitudinal studies with repeated urine sampling.

The predominance of CLO, DIN, THM, and DM-ACE in the present study may reflect the combined effects of geographic setting, regional pesticide use patterns, dietary sources, and compound-specific metabolism. A recent Guangzhou market-basket study reported that vegetables were the dominant contributor to cumulative dietary NEO exposure, with leafy vegetables mainly contributing to THM and ACE intake and rice contributing substantially to CLO and DIN exposure [48]. Consistently, a sediment study in South China found that NEO profiles varied by crop type, with CLO and IMI dominating in rice-planting areas and ACE and THD more frequently detected in vegetable-planting areas [49]. In addition, environmental monitoring studies have shown that IMI, CLO, THM, and ACE are dominant NEOs in surface waters of South China, suggesting that local pesticide-use patterns, hydrological transport, and agricultural runoff may shape regional exposure signatures [50]. Evidence from Shenzhen also showed frequent ACE residues in vegetables and fruits, which may help explain the high urinary contribution of DM-ACE, the major metabolite of ACE [47]. Thus, the heterogeneous composition profiles across centers may represent different exposure signatures: DIN- and CLO/THM-enriched profiles could indicate greater contributions from rice- or vegetable-related exposure and local environmental inputs, whereas DM-ACE/IMI-OF enrichment may reflect a stronger contribution from metabolite-based exposure markers. Such composition analysis provides useful clues for source inference, especially in the absence of direct dietary and environmental measurements. However, this interpretation requires confirmation in future studies with direct dietary and environmental data.

The correlation analysis further demonstrated that older adults were simultaneously exposed to multiple NEOs. Particularly strong correlations were observed between THM and CLO, as well as between IMI and its metabolites. The close association between THM and CLO is biologically plausible because CLO can be formed as a metabolite of THM in environmental and biological systems [2,51]. The correlations between IMI and its metabolites, 5-OH-IMI and IMI-OF, likely reflect parent–metabolite relationships commonly used in NEO biomonitoring [12]. These results indicate that urinary NEO profiles represent mixture exposure rather than isolated single-compound exposure, highlighting the need to consider co-exposure patterns in future health-risk assessments.

Another finding was the observed association between several urinary NEO biomarkers and metabolic or liver function indicators. DM-THM showed the most consistent associations with GLU, HbA1c, TG, and ALT. Although causality cannot be inferred due to the cross-sectional design, these findings are broadly consistent with previous epidemiological evidence linking NEO exposure to cardiometabolic and endocrine outcomes [3,27,28,33]. However, some observed correlation coefficients were weak, and most nominal associations did not survive FDR correction. Therefore, these results should be considered exploratory and hypothesis-generating. Mechanistically, experimental studies suggest that NEOs may induce oxidative stress, mitochondrial dysfunction, and insulin signaling disruption [25,52,53]. Nevertheless, most evidence comes from high-dose animal or in vitro models, and its relevance to low-level environmental exposure remains unclear; thus, these potential pathways require confirmation in future longitudinal and mechanistic studies.

Several limitations should be considered when interpreting these findings. First, the cross-sectional design precludes causal inference, and all exposure-outcome associations should be regarded as exploratory and hypothesis-generating. Second, NEOs and their metabolites have short biological half-lives, typically on the order of hours, so a single first morning spot urine sample mainly reflects recent exposure and may not represent long-term or usual exposure. Third, first morning urine samples were collected in this study, which are less susceptible to short-term fluid intake and diurnal variation, and are generally considered more reproducible and representative than random spot samples for biomonitoring purposes [54,55]. However, residual confounding by hydration status cannot be completely ruled out, and comparisons with studies that applied creatinine- or specific gravity adjustment should therefore be made with caution. Fourth, detailed dietary and occupational information was not collected, which limited our ability to identify specific exposure sources and to fully control for relevant confounders.

## 5. Conclusions

In summary, this study demonstrated widespread co-exposure to multiple NEOs among older adults in South China. The predominance of CLO, DIN, THM, and DM-ACE, along with exploratory associations with glucose, lipid, uric acid, and liver function-related indicators, highlights the need for continued biomonitoring and further investigation of the potential health implications of chronic low-level NEO exposure in aging populations. Given the cross-sectional design, these findings are preliminary. Future longitudinal studies incorporating repeated biospecimen collection, dietary assessment, environmental monitoring, and mechanistic biomarkers are warranted to clarify exposure sources, temporal variability, and potential pathways linking NEO exposure to metabolic and age-related health outcomes.

## Figures and Tables

**Table 1:** Participant characteristics of older adults from Guangzhou and Shenzhen.

Characteristic	Total (n = 419)	Guangzhou (n = 140)	Shenzhen (n = 279)	*p* Value
**Sex (n, %)**				0.532
**Female**	196 (46.8)	69 (49.3)	127 (45.5)	
**Male**	223 (53.2)	71 (50.7)	152 (54.5)	
**Smoke (n, %)**				**<0.001**
**No**	333 (79.5)	117 (83.6)	216 (77.4)	
**Yes**	79 (18.9)	16 (11.4)	63 (22.6)	
**Alcohol (n, %)**				**<0.001**
**No**	373 (89.0)	132 (94.3)	241 (86.4)	
**Yes**	42 (10.0)	4 (2.9)	38 (13.6)	
**Hypertension (n, %)**				**0.004**
**No**	283 (67.5)	108 (77.1)	175 (62.7)	
**Yes**	136 (32.5)	32 (22.9)	104 (37.3)	
**Age (median, IQR)**	66.00 (63.00, 72.00)	65.00 (62.00, 70.00)	68.00 (64.00, 73.00)	**<0.001**
**GLU (median, IQR)**	5.36 (4.76, 7.04)	5.10 (4.66, 5.66)	5.61 (4.90, 7.37)	**<0.001**
**HbA1c (median, IQR)**	5.80 (5.50, 6.30)	5.70 (5.30, 6.20)	5.90 (5.60, 6.40)	**<0.001**
**SUA (median, IQR)**	358.00 (294.08, 437.97)	341.65 (272.05, 426.35)	371.00 (304.50, 438.40)	**0.047**
**SCr (median, IQR)**	73.00 (59.80, 85.46)	75.49 (65.00, 89.25)	72.20 (58.35, 84.00)	**0.011**
**TC (median, IQR)**	4.67 (3.90, 5.59)	4.69 (3.98, 5.49)	4.66 (3.77, 5.63)	0.707
**TG (median, IQR)**	1.30 (0.95, 1.74)	1.08 (0.78, 1.47)	1.38 (1.06, 1.90)	**<0.001**
**LDL (median, IQR)**	2.85 (2.34, 3.59)	2.88 (2.43, 3.56)	2.85 (2.26, 3.59)	0.359
**HDL (median, IQR)**	1.31 (1.12, 1.50)	1.29 (1.07, 1.47)	1.33 (1.14, 1.51)	0.168
**ALT (median, IQR)**	18.48 (13.00, 27.00)	16.02 (11.75, 23.00)	20.76 (14.11, 28.74)	**<0.001**
**AST (median, IQR)**	22.00 (18.00, 27.00)	21.00 (18.00, 25.67)	22.00 (18.68, 27.79)	0.088

Notes: Continuous variables are presented as median (interquartile range, IQR), and categorical variables are presented as number (percentage). Differences between Guangzhou and Shenzhen were assessed using the Mann–Whitney U test for continuous variables and the chi-square test or Fisher’s exact test for categorical variables, as appropriate. n, number; GLU, fasting blood glucose (mmol/L); HbA1c, glycated hemoglobin (%); SUA, serum uric acid (μmol/L); SCr, serum creatinine (μmol/L); TC, total cholesterol (mmol/L); TG, triglycerides (mmol/L); LDL, low-density lipoprotein cholesterol (mmol/L); HDL, high-density lipoprotein cholesterol (mmol/L); ALT, alanine aminotransferase (U/L); AST, aspartate aminotransferase (U/L).

**Table 2:** Detection frequencies and urinary concentrations of NEOs and metabolites (μg/L).

Compound	DF (%)	Mean (SD)	GM (GSD)	Median (IQR)	P95
**THD**	81.67	0.035 (0.067)	0.018 (2.966)	0.018 (0.008, 0.037)	0.105
**THM**	99.76	2.006 (4.408)	0.847 (3.628)	0.888 (0.357, 1.971)	6.506
**CLO**	99.05	2.836 (5.057)	1.360 (3.421)	1.402 (0.641, 3.067)	9.318
**IMI**	97.38	0.345 (0.469)	0.190 (2.909)	0.155 (0.102, 0.382)	1.458
**ACE**	65.48	0.065 (0.148)	0.030 (2.877)	0.025 (0.010, 0.054)	0.245
**DIN**	99.29	3.161 (6.929)	1.164 (4.257)	1.240 (0.471, 2.969)	12.149
**5-OH-IMI**	98.81	1.904 (7.254)	0.775 (3.815)	0.820 (0.382, 1.745)	5.710
**IMI-OF**	95.48	1.799 (3.063)	0.700 (4.587)	0.826 (0.297, 1.860)	6.300
**DM-ACE**	99.76	2.957 (5.844)	1.568 (3.123)	1.643 (0.798, 3.239)	9.936
**DM-THM**	92.14	0.497 (1.768)	0.194 (3.319)	0.184 (0.082, 0.446)	1.356
**ΣNEOs**	-	15.607 (16.676)	10.822 (2.360)	10.928 (6.170, 19.620)	41.624

Notes: Values below the limit of detection were replaced with LOD/√2. ΣNEOs was calculated as the sum of the measured parent NEOs and metabolites after replacing values below the LOD with LOD/√2. DF, detection frequency; SD, standard deviation; GM, geometric mean; GSD, geometric standard deviation; IQR, interquartile range; P95, 95th percentile.

**Table 3:** Comparison of median urinary levels of NEOs and metabolites reported in different populations.

Country	Population	Sample Size	Unit	THD	THM	CLO	IMI	ACE	DIN	5-OH-IMI	IMI-OF	DM-ACE	DM-THM	Reference
South China	elderly adults	1147	μg/L	0.02	0.53	0.80	0.13	0.03	1.31	1.91	2.21	2.74	0.22	[3]
South China	children	88	μg/g Cr	2.7	28	60	11	1.2	17	29	42	89	-	[28]
South China	adults	225	μg/L	0.024	0.90	1.23	0.14	0.012	0.20	0.71	0.57	1.31	-	[25]
Ireland	farm and non-farm families	227	μg/L	<LOQ	<LOQ	<LOQ	<LOQ	<LOQ	-	<LOQ	<LOQ	0.21	<LOQ	[40]
South China	neonates	92	μg/L (μg/g Cr)	<LOD	<LOD	0.154 (0.272)	<LOD	<LOD	-	-	<LOD	0.291 (0.444)	<LOD	[41]
East China	pregnant women	432	μg/L (μg/g Cr)	<LOD	<LOD	0.45 (0.32)	<LOD	<LOD	<LOD	<LOD	-	1.31 (0.88)	<LOD	[37]
Tibet, China	pregnant women	476	μg/L	<LOD (0.07)	<LOD (0.02)	<LOD (0.007)	<LOD (0.005)	<LOD (0.07)	<LOD (0.08)	<LOD (0.11)	-	<LOD (0.01)	<LOD (0.05)	[38]
Philippines	Rural population	99	μg/L	<LOQ	<LOQ	<LOQ	<LOQ	<LOQ	<LOQ	-	-	<LOQ	-	[39]

Notes: Values in parentheses indicate creatinine-adjusted concentrations when reported by the original study. “-” indicates that the compound was not measured or not reported. LOD, limit of detection; LOQ, limit of quantification; Cr, creatinine. Because the included studies reported urinary concentrations using different normalization approaches, including volume-based concentrations and creatinine-adjusted concentrations, direct quantitative comparisons across studies should be interpreted cautiously.

## Data Availability

Data are available from the corresponding author by request.

## Supplementary Materials

The following supporting information can be downloaded at: https://www.mdpi.com/article/10.3390/toxics14070641/s1, Text S1: Sample pre-treatment and instrumental analysis of NEOs; Text S2: Quality control and quality assurance; Table S1: Recruitment centers and sample size distribution; Table S2: Information of the analytes for mass spectrometry; Table S3: Quality control results; Table S4: Spearman correlations between urinary NEO biomarkers and continuous variables; Table S5: Associations between categorical participant characteristics and urinary NEO biomarkers based on multivariable linear regression models.

## Abbreviations

**Abbreviation**	**Full term**
NEOs	Neonicotinoid insecticides
THD	Thiacloprid
THM	Thiamethoxam
CLO	Clothianidin
IMI	Imidacloprid
ACE	Acetamiprid
DIN	Dinotefuran
5-OH-IMI	5-Hydroxy-imidacloprid
IMI-OF	Imidacloprid-olefin
DM-ACE	*N*-desmethyl-acetamiprid
DM-THM	*N*-desmethyl-thiamethoxam
GLU	Fasting blood glucose
HbA1c	Glycated hemoglobin
SUA	Serum uric acid
SCr	Serum creatinine
TC	Total cholesterol
TG	Triglycerides
LDL	Low-density lipoprotein cholesterol
HDL	High-density lipoprotein cholesterol
ALT	Alanine aminotransferase
AST	Aspartate aminotransferase
LODs	limits of detection
DF	Detection frequency
IQRs	Interquartile ranges
SDs	Standard deviations
GSDs	Geometric standard deviations
P95	95th percentile
