# Supplementary material for: Uncovering Urinary Neonicotinoid Exposure Signatures Among Older Adults in South China: A Multicenter Biomonitoring Study
Source: Toxics. 2026 Jul 22;14(7):641. doi: 10.3390/toxics14070641 (PMC13418382; doi:10.3390/toxics14070641)
Supplement: Supplementary file 1 [file toxics-14-00641-s001.zip › toxics-4419915-supplementary.pdf]

Uncovering Urinary Neonicotinoid Exposure Signatures Among Older Adults in South China: A Multicenter Biomonitoring Study

Xiaoxiao Chen [1,†], Chiqun Shan [2,†], Yiming Ge [2], Yuli Lin [2], Bo Fu [2], Canrong Zheng [1], Yuhua Huang [1,*] and Shaoyou Lu [2,*]

[1] Chaozhou People's Hospital, Chaozhou Hospital Affiliated to Shantou University Medical College, Chaozhou 521011, China; 15992360101@163.com (X.C.); 13715821998@163.com (C.Z.)

[2] Research Center for Environment and Health, School of Public Health (Shenzhen), Sun Yat-sen University, Shenzhen 518107, China; shanchq3@mail2.sysu.edu.cn (C.S.); geym5@mail2.sysu.edu.cn (Y.G.); linyli6@mail2.sysu.edu.cn (Y.L.); fubo7@mail2.sysu.edu.cn (B.F.)

* Correspondence: hyh730420@sina.com (Y.H.); lushy23@mail.sysu.edu.cn (S.L.)

† These authors contributed equally to this work.

Text S1 Sample pre-treatment and instrumental analysis of NEOs

A liquid–liquid partition method was employed for NEOs. Briefly, 1 mL of the urine sample was transferred into a 15 mL glass centrifuge tube, to which 20 μL of internal standard mixture (100 ng/mL) was added. The sample was then combined with 1 mL of 1 mol/L ammonium acetate buffer solution and 20 μL of β-glucuronidase (aqueous solution, > 100,000 units/mL), thoroughly mixed, and incubated at 37°C for 12 hours. The target analytes were subsequently extracted three times with ethyl acetate (10 mL), and the mixture was shaken vigorously for 10 minutes, followed by centrifugation at 3000 rpm/min for 10 minutes. The supernatants were combined and concentrated to near-dryness under nitrogen. The residue was reconstituted with 0.5 mL of methanol, filtered through a 0.22 μm membrane (Anpel, Shanghai, China), and stored at -20°C until instrumental analysis. NEOs were separated using a 20A high-performance liquid chromatography (HPLC) system (Shimadzu, Kyoto, Japan) coupled with an AB SCIEX Q-trap 6500 triple quadrupole mass spectrometer (MS/MS, Applied Biosystems, Waltham, MA, USA) for quantification. The separation was performed on an ACQUITY UPLC HSS T3 column (2.1 × 100 mm, 1.8 μm, Waters, Ireland). The mobile phase was 0.1% formic acid in water (A) and acetonitrile (B). The flow rate was set at 0.3 mL/min. The mobile phase gradients were programmed as follows: 0-3 min, 99%-70% solvent A; 3-6 min, 70%-55% solvent A; 6-6.1 min, 55%-99% solvent A; 6.1-7 min, 99% solvent A. Positive electrospray ionization (ESI+) was carried out.

Text S2 Quality control and quality assurance

All glassware used in the experiments was baked at 450°C to eliminate contaminants. Procedural and instrumental blanks were analyzed for each batch of samples (n = 14) to evaluate

background contamination. Calibration curves were constructed using seven concentration levels (0, 1, 5, 10, 25, 50, and 100 μg/L) to quantify the target analytes, with all R^2 values exceeding 0.995 (Table S3). Intra- and inter-batch relative standard deviation (RSD) values were calculated from duplicate and spiked samples, respectively. All RSD values were within 10%, and matrix spike recoveries ranged from 80% to 120%. The limit of detection (LOD) varied from 0.0055 to 0.0441 μg/L, and the limits of quantification (LOQ) ranged from 0.0182 to 0.1471 μg/L (Table S3).

Table S1. Recruitment centers and sample size distribution.

Center ID	City	Institution type	Sample size
Center 1	Guangzhou	Hospital	77
Center 2	Guangzhou	Hospital	63
Center 3	Shenzhen	Community health service center	93
Center 4	Shenzhen	Hospital	90
Center 5	Shenzhen	Community health service center	96

Table S2 Information of the analytes for mass spectrometry.

Analyte	Internal standard	Parent ion (m/z)	Product ion (m/z)		CE (V)		DP (V)
THD	THD-D4	253.2	126.3	90.2	28	50	50
THM	THM-D3	292	211.1	132.2	16	23	30
CLO	CLO-D3	250.1	169.1	132.2	13	16	20
IMI	IMI-D4	255.9	209.1	175.3	21	23	30
ACE	ACE-D3	223	126.1	56.1	26	19	40
DIN	DIN-D3	203.1	129	114	15	19	20
5-OH-IMI	IMI-D4	272	191.1	225.1	22	24	40
IMI-OF	IMI-D4	254	205	171.1	21	23	30
DM-ACE	ACE-D3	209	126.2	90	21	38	40
DM-THM	THM-D3	278	132.1	197.2	24	20	30
THD-D4		257.2	126	190.1	27	20	30
THM-D3		295.2	214.1	132.2	16	24	30
CLO-D3		253.1	172.2	132	14	19	30
IMI-D4		260.1	213.1	179.2	19	23	30
ACE-D3		226.1	126.1	59	27	19	40
DIN-D3		206.1	132.3	90.3	16	20	30

CE: Collision Energy.

DP: Declustering Potential.

Table S3 Quality control results.

Analyte	Standard curve	R^2	LOD (μg/L)	LOQ (μg/L)	RSD (%)	Matrix spike recovery (%)
THD	y=0.78738x+0.00168	0.995	0.0055	0.0182	2.5	111
THM	y=0.96374x+8.19985e^{-4}	0.996	0.0141	0.0471	8.5	95
CLO	y=0.86583x+5.96638e^{-4}	0.996	0.0441	0.1471	2.5	92
IMI	y=0.95656x+7.64720e^{-4}	0.997	0.0255	0.085	2.2	94
ACE	y=0.95725x+0.00134	0.996	0.0156	0.0519	2.3	96
DIN	y=0.86612x+0.01224	0.998	0.0075	0.0251	6.8	101
5-OH-IMI	y=0.56443x+0.00143	0.997	0.0089	0.0296	1.7	81
IMI-OF	y=0.08554x+0.00152	0.996	0.0228	0.076	5.3	82
DM-ACE	y=0.88501x+0.00189	0.998	0.041	0.1366	2.9	110
DM-THM	y=0.71178x+0.00342	0.996	0.0366	0.122	9.4	119

Notes: LOD: limit of detection. LOQ: limit of quantification. RSD: Relative standard deviation. All concentrations are expressed in μg/L.

Table S4 Spearman correlations between urinary NEO biomarkers and continuous variables.

Variable	THD	THM	CLO	IMI	ACE	DIN	5-OH-IMI	IMI-OF	DM-ACE	DM-THM
**Age**	0.028	-0.034	-0.105	-0.011	-0.033	-0.013	-0.015	-0.003	-0.036	0.096
	P = 0.563, q = 0.924	P = 0.491, q = 0.906	P = 0.031, q = 0.287	P = 0.823, q = 0.983	P = 0.506, q = 0.906	P = 0.788, q = 0.983	P = 0.756, q = 0.983	P = 0.951, q = 0.983	P = 0.460, q = 0.906	P = 0.050, q = 0.394
**GLU**	0.048	0.092	0.080	0.014	0.047	0.070	0.021	-0.008	0.068	0.253
	P = 0.326, q = 0.882	P = 0.060, q = 0.436	P = 0.101, q = 0.555	P = 0.781, q = 0.983	P = 0.334, q = 0.882	P = 0.151, q = 0.663	P = 0.668, q = 0.967	P = 0.877, q = 0.983	P = 0.164, q = 0.670	**P < 0.001, q < 0.001**
**HbA1c**	0.115	0.002	0.025	-0.047	0.018	0.032	0.002	0.048	0.047	0.188
	P = 0.019, q = 0.257	P = 0.975, q = 0.983	P = 0.613, q = 0.942	P = 0.334, q = 0.882	P = 0.721, q = 0.983	P = 0.511, q = 0.906	P = 0.961, q = 0.983	P = 0.322, q = 0.882	P = 0.335, q = 0.882	***P* < 0.001, *q* = 0.003**
**SUA**	0.056	-0.003	-0.001	0.144	0.091	0.107	0.043	0.081	-0.050	0.115
	P = 0.255, q = 0.860	P = 0.948, q = 0.983	P = 0.978, q = 0.983	P = 0.003, q = 0.057	P = 0.063, q = 0.436	P = 0.029, q = 0.287	P = 0.380, q = 0.906	P = 0.098, q = 0.555	P = 0.311, q = 0.882	P = 0.018, q = 0.257
**SCr**	0.040	-0.002	-0.022	-0.036	-0.037	0.051	0.004	-0.012	0.008	-0.007
	P = 0.418, q = 0.906	P = 0.969, q = 0.983	P = 0.649, q = 0.965	P = 0.465, q = 0.906	P = 0.447, q = 0.906	P = 0.296, q = 0.882	P = 0.939, q = 0.983	P = 0.808, q = 0.983	P = 0.865, q = 0.983	P = 0.890, q = 0.983
**TC**	-0.009	-0.014	0.035	0.034	0.009	0.006	0.020	0.044	-0.038	0.007
	P = 0.858, q = 0.983	P = 0.776, q = 0.983	P = 0.472, q = 0.906	P = 0.489, q = 0.906	P = 0.851, q = 0.983	P = 0.901, q = 0.983	P = 0.680, q = 0.971	P = 0.370, q = 0.903	P = 0.444, q = 0.906	P = 0.882, q = 0.983
**TG**	0.031	0.006	0.033	0.048	0.045	0.059	0.071	0.038	-0.037	0.192
	P = 0.528, q = 0.907	P = 0.897, q = 0.983	P = 0.495, q = 0.906	P = 0.328, q = 0.882	P = 0.361, q = 0.903	P = 0.229, q = 0.811	P = 0.147, q = 0.663	P = 0.437, q = 0.906	P = 0.452, q = 0.906	***P* < 0.001, *q* = 0.003**
**LDL**	-0.017	-0.014	0.047	0.002	0.002	-0.016	-0.026	0.001	-0.044	-0.017
	P = 0.731, q = 0.983	P = 0.773, q = 0.983	P = 0.337, q = 0.882	P = 0.960, q = 0.983	P = 0.960, q = 0.983	P = 0.739, q = 0.983	P = 0.599, q = 0.941	P = 0.983, q = 0.983	P = 0.365, q = 0.903	P = 0.723, q = 0.983
**HDL**	0.008	0.082	0.156	0.069	-0.030	0.022	0.074	0.029	0.026	0.070
	P = 0.866, q = 0.983	P = 0.095, q = 0.555	***P* = 0.001, *q* = 0.030**	P = 0.157, q = 0.663	P = 0.537, q = 0.909	P = 0.650, q = 0.965	P = 0.133, q = 0.663	P = 0.553, q = 0.921	P = 0.597, q = 0.941	P = 0.150, q = 0.663
**ALT**	0.105	0.002	-0.025	-0.001	0.067	0.079	0.065	0.036	0.032	0.202
	P = 0.031, q = 0.287	P = 0.969, q = 0.983	P = 0.616, q = 0.942	P = 0.976, q = 0.983	P = 0.172, q = 0.671	P = 0.108, q = 0.563	P = 0.185, q = 0.678	P = 0.459, q = 0.906	P = 0.520, q = 0.907	P < 0.001, q = 0.002
**AST**	0.039	0.034	-0.005	0.027	0.099	0.087	0.055	0.022	0.066	0.110
	P = 0.428, q = 0.906	P = 0.488, q = 0.906	P = 0.923, q = 0.983	P = 0.584, q = 0.941	P = 0.043, q = 0.365	P = 0.075, q = 0.487	P = 0.258, q = 0.860	P = 0.658, q = 0.965	P = 0.177, q = 0.671	P = 0.025, q = 0.287

Notes: Values are Spearman correlation coefficients (r). P = P-value, q = FDR-adjusted q-value. Urinary NEO concentrations were transformed before analysis.

Table S5 Associations between categorical participant characteristics and urinary NEO biomarkers based on multivariable linear regression models.

Variable	THD	THM	CLO	IMI	ACE	DIN	5-OH-IMI	IMI-OF	DM-ACE	DM-THM
**Sex**	0.140 *P* = 0.246, *q* = 0.746	-0.050 *P* = 0.723, *q* = 0.908	-0.135 *P* = 0.318, *q* = 0.765	0.026 *P* = 0.826, *q* = 0.944	0.029 *P* = 0.807, *q* = 0.944	0.102 *P* = 0.524, *q* = 0.776	-0.112 *P* = 0.454, *q* = 0.775	0.166 *P* = 0.325, *q* = 0.765	-0.008 *P* = 0.951, *q* = 0.975	0.216 *P* = 0.102, *q* = 0.554
**Smoking**	-0.054 *P* = 0.715, *q* = 0.908	-0.017 *P* = 0.924, *q* = 0.972	0.116 *P* = 0.484, *q* = 0.775	-0.201 *P* = 0.168, *q* = 0.611	-0.096 *P* = 0.503, *q* = 0.775	-0.404 *P* = 0.040, *q* = 0.463	-0.024 *P* = 0.894, *q* = 0.972	-0.385 *P* = 0.063, *q* = 0.463	-0.126 *P* = 0.418, *q* = 0.775	-0.078 *P* = 0.630, *q* = 0.869
**Alcohol consumption**	0.172 *P* = 0.261, *q* = 0.746	-0.328 *P* = 0.069, *q* = 0.463	-0.184 *P* = 0.283, *q* = 0.755	0.129 *P* = 0.389, *q* = 0.775	0.088 *P* = 0.552, *q* = 0.789	0.317 *P* = 0.119, *q* = 0.554	0.289 *P* = 0.125, *q* = 0.554	0.468 *P* = 0.029, *q* = 0.463	0.144 *P* = 0.369, *q* = 0.775	0.044 *P* = 0.791, *q* = 0.944
**Hypertension**	0.102 *P* = 0.372, *q* = 0.775	0.095 *P* = 0.483, *q* = 0.775	0.153 *P* = 0.236, *q* = 0.746	-0.003 *P* = 0.978, *q* = 0.978	0.216 *P* = 0.054, *q* = 0.463	0.223 *P* = 0.144, *q* = 0.574	-0.049 *P* = 0.727, *q* = 0.908	-0.125 *P* = 0.436, *q* = 0.775	-0.013 *P* = 0.917, *q* = 0.972	0.299 *P* = 0.018, *q* = 0.463

Notes: Values are multivariable linear regression coefficients (β). *P* = *P*-value, *q* = FDR-adjusted *q*-value. Regression coefficients from multivariable linear models assessing associations between categorical participant characteristics and log-transformed urinary NEO concentrations, adjusted for age, sex, smoking status, alcohol consumption, hypertension status, study center and SCr.
